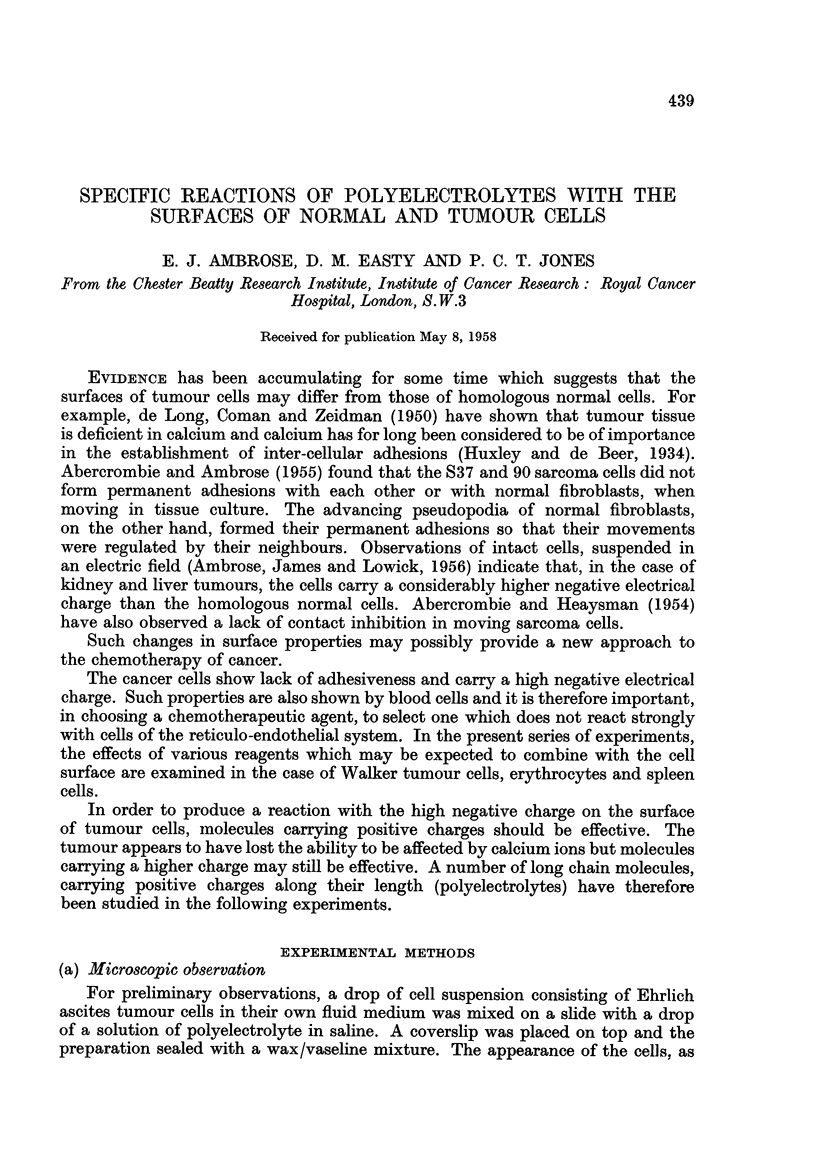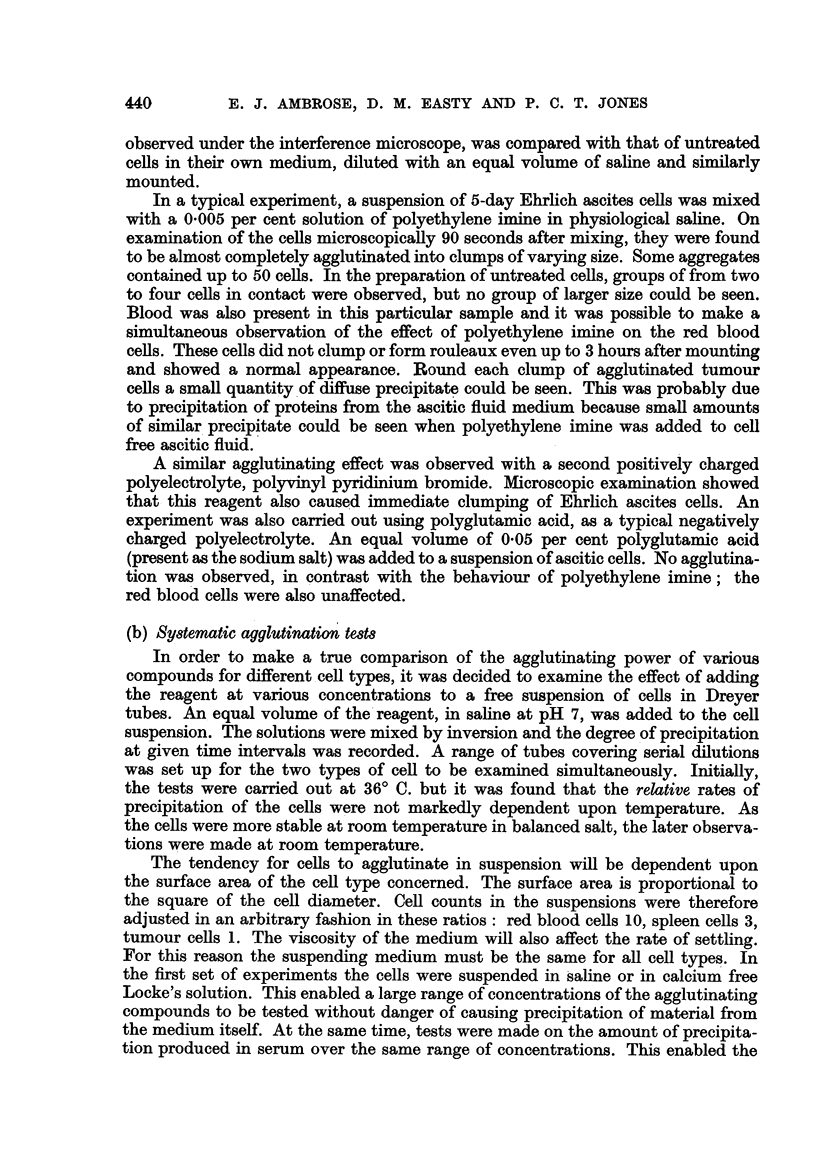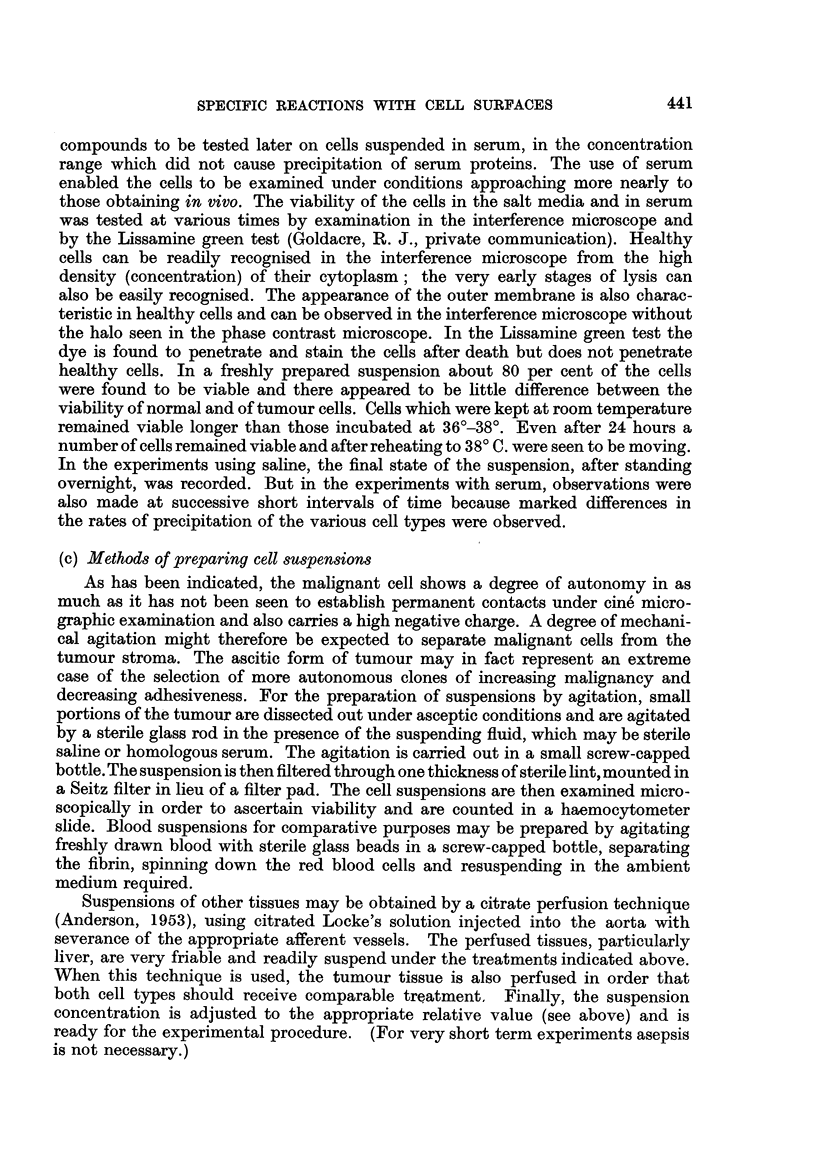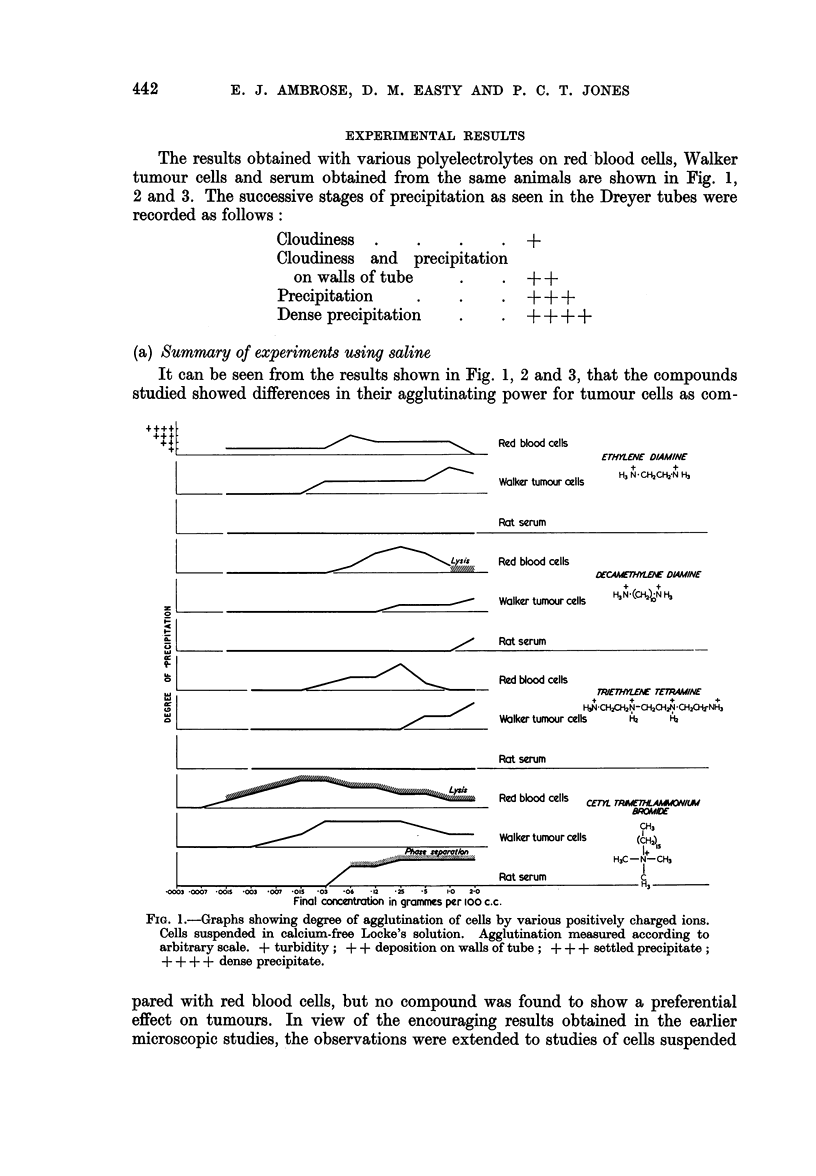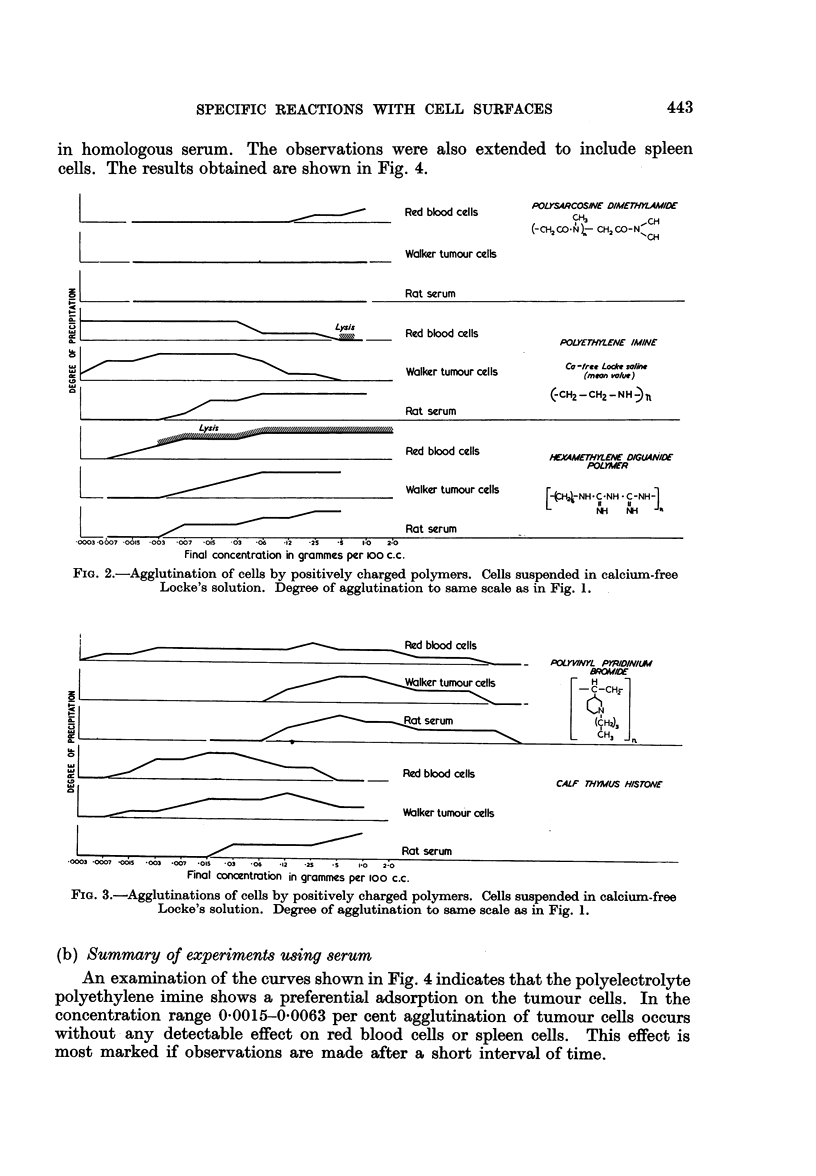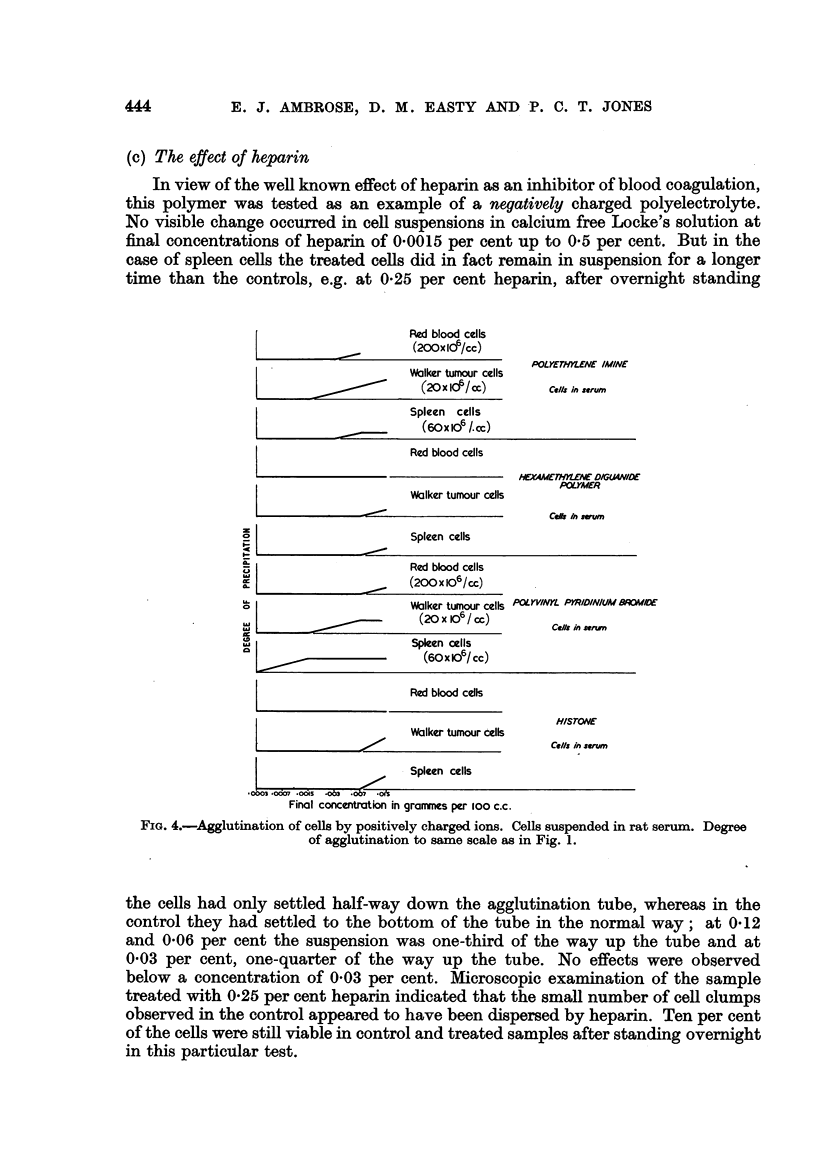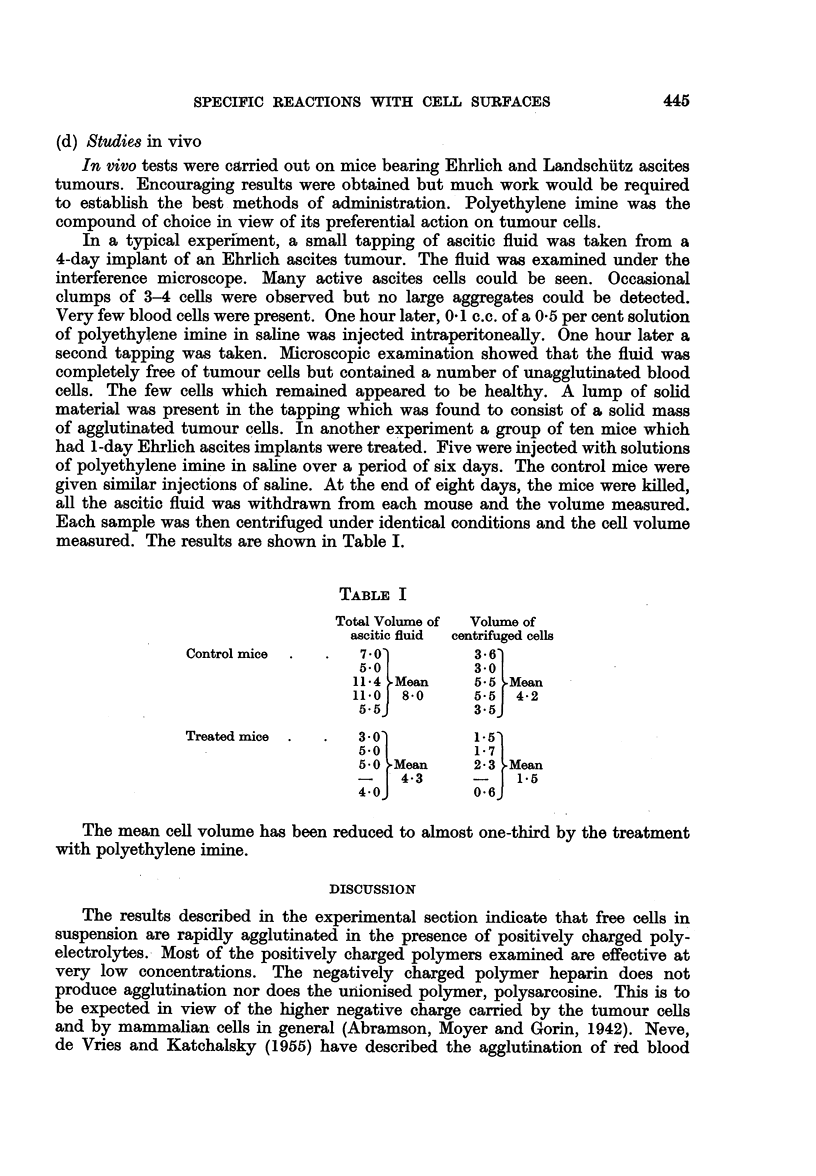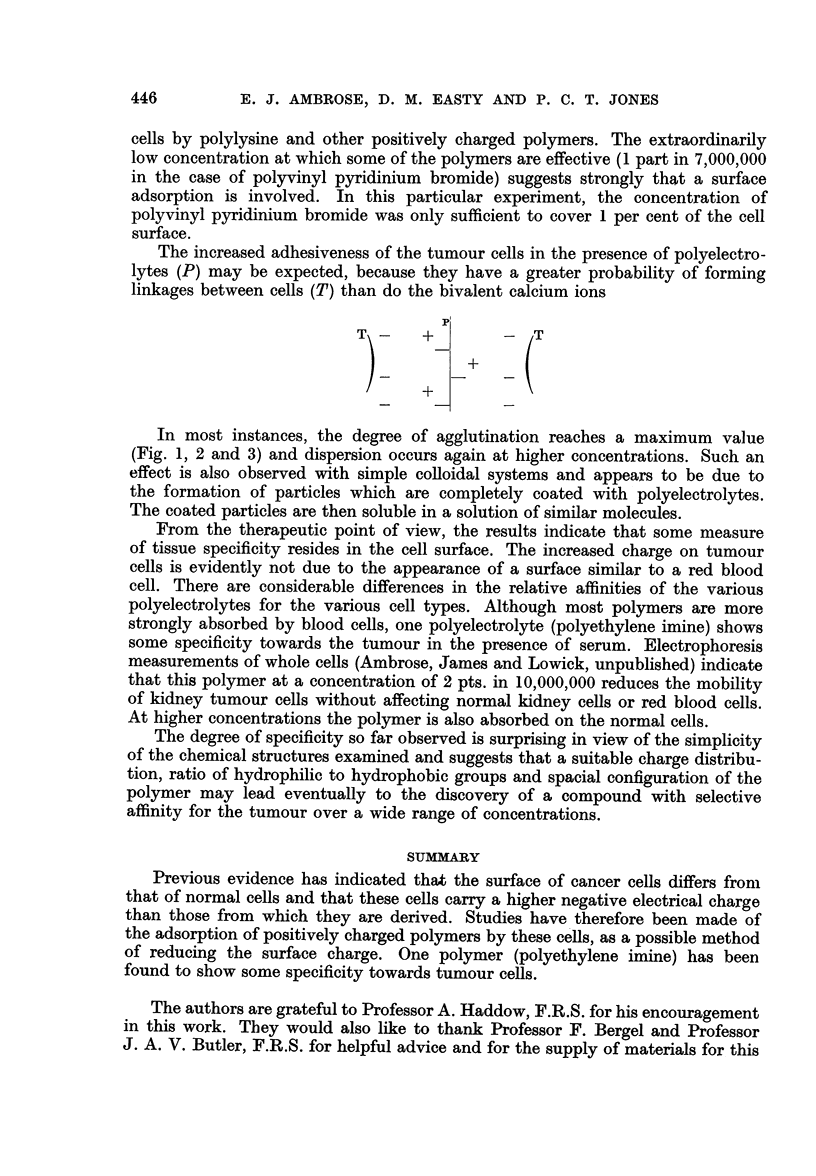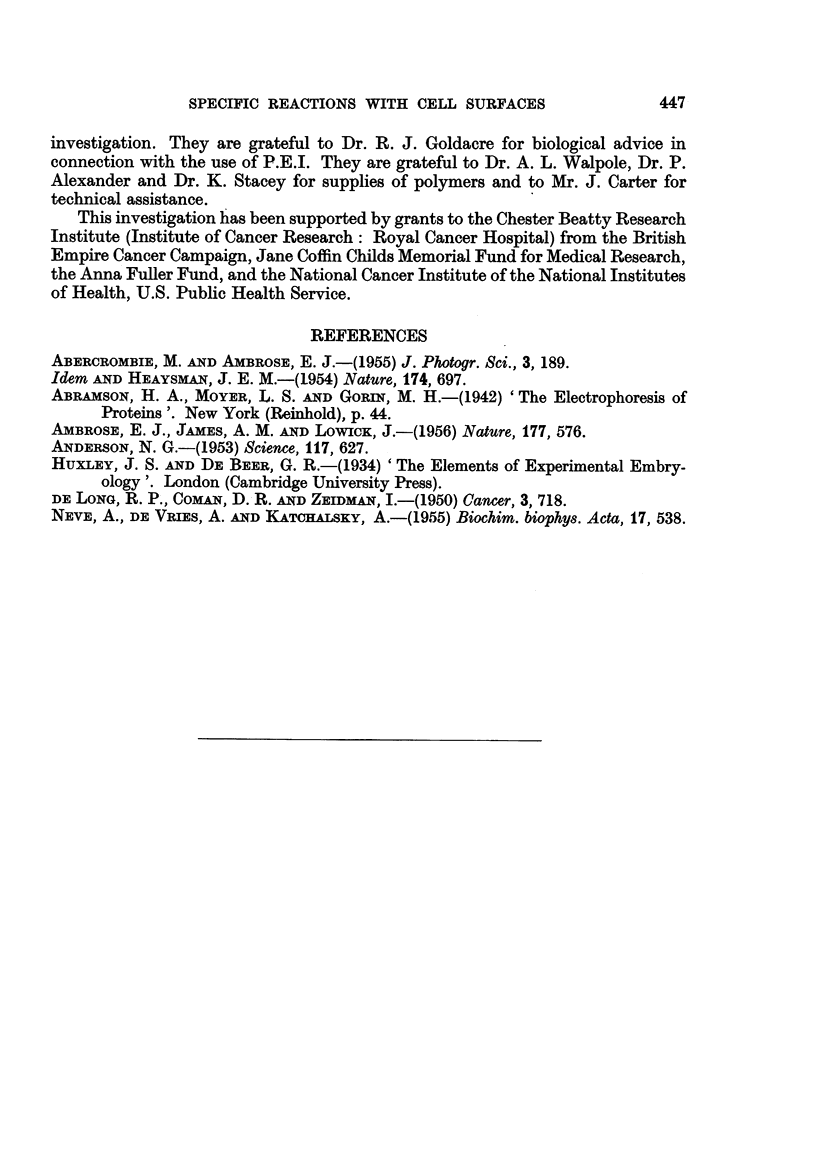# Specific Reactions of Polyelectrolytes with the Surfaces of Normal and Tumour Cells

**DOI:** 10.1038/bjc.1958.52

**Published:** 1958-09

**Authors:** E. J. Ambrose, D. M. Easty, P. C. T. Jones


### SPECIFIC REACTIONS OF POLYELECTROLYTES WITH THE SURFACES OF NORMAL AND TUMOUR CELLS

E. J. AMBROSE, D. M. EASTY AND P. C. T. JONES

*From the Chester Beatty Research Institute, Institute of Cancer Research : Royal Cancer Hospital, London, S.W.3*

Received for publication May 8, 1958

EVIDENCE has been accumulating for some time which suggests that the surfaces of tumour cells may differ from those of homologous normal cells. For example, de Long, Coman and Zeidman (1950) have shown that tumour tissue is deficient in calcium and calcium has for long been considered to be of importance in the establishment of inter-cellular adhesions (Huxley and de Beer, 1934). Abercrombie and Ambrose (1955) found that the S37 and 90 sarcoma cells did not form permanent adhesions with each other or with normal fibroblasts, when moving in tissue culture. The advancing pseudopodia of normal fibroblasts, on the other hand, formed their permanent adhesions so that their movements were regulated by their neighbours. Observations of intact cells, suspended in an electric field (Ambrose, James and Lowick, 1956) indicate that, in the case of kidney and liver tumours, the cells carry a considerably higher negative electrical charge than the homologous normal cells. Abercrombie and Heaysman (1954) have also observed a lack of contact inhibition in moving sarcoma cells.

Such changes in surface properties may possibly provide a new approach to the chemotherapy of cancer.

The cancer cells show lack of adhesiveness and carry a high negative electrical charge. Such properties are also shown by blood cells and it is therefore important, in choosing a chemotherapeutic agent, to select one which does not react strongly with cells of the reticulo-endothelial system. In the present series of experiments, the effects of various reagents which may be expected to combine with the cell surface are examined in the case of Walker tumour cells, erythrocytes and spleen cells.

In order to produce a reaction with the high negative charge on the surface of tumour cells, molecules carrying positive charges should be effective. The tumour appears to have lost the ability to be affected by calcium ions but molecules carrying a higher charge may still be effective. A number of long chain molecules, carrying positive charges along their length (polyelectrolytes) have therefore been studied in the following experiments.

### EXPERIMENTAL METHODS

### (a) *Microscopic observation*

For preliminary observations, a drop of cell suspension consisting of Ehrlich ascites tumour cells in their own fluid medium was mixed on a slide with a drop of a solution of polyelectrolyte in saline. A coverslip was placed on top and the preparation sealed with a wax/vaseline mixture. The appearance of the cells, as observed under the interference microscope, was compared with that of untreated cells in their own medium, diluted with an equal volume of saline and similarly mounted.

In a typical experiment, a suspension of 5-day Ehrlich ascites cells was mixed with a 0·005 per cent solution of polyethylene imine in physiological saline. On examination of the cells microscopically 90 seconds after mixing, they were found to be almost completely agglutinated into clumps of varying size. Some aggregates contained up to 50 cells. In the preparation of untreated cells, groups of from two to four cells in contact were observed, but no group of larger size could be seen. Blood was also present in this particular sample and it was possible to make a simultaneous observation of the effect of polyethylene imine on the red blood cells. These cells did not clump or form rouleaux even up to 3 hours after mounting and showed a normal appearance. Round each clump of agglutinated tumour cells a small quantity of diffuse precipitate could be seen. This was probably due to precipitation of proteins from the ascitic fluid medium because small amounts of similar precipitate could be seen when polyethylene imine was added to cell free ascitic fluid.

A similar agglutinating effect was observed with a second positively charged polyelectrolyte, polyvinyl pyridinium bromide. Microscopic examination showed that this reagent also caused immediate clumping of Ehrlich ascites cells. An experiment was also carried out using polyglutamic acid, as a typical negatively charged polyelectrolyte. An equal volume of 0·05 per cent polyglutamic acid (present as the sodium salt) was added to a suspension of ascitic cells. No agglutination was observed, in contrast with the behaviour of polyethylene imine; the red blood cells were also unaffected.

(b) *Systematic agglutination tests*

In order to make a true comparison of the agglutinating power of various compounds for different cell types, it was decided to examine the effect of adding the reagent at various concentrations to a free suspension of cells in Dreyer tubes. An equal volume of the reagent, in saline at pH 7, was added to the cell suspension. The solutions were mixed by inversion and the degree of precipitation at given time intervals was recorded. A range of tubes covering serial dilutions was set up for the two types of cell to be examined simultaneously. Initially, the tests were carried out at 36° C. but it was found that the *relative* rates of precipitation of the cells were not markedly dependent upon temperature. As the cells were more stable at room temperature in balanced salt, the later observations were made at room temperature.

The tendency for cells to agglutinate in suspension will be dependent upon the surface area of the cell type concerned. The surface area is proportional to the square of the cell diameter. Cell counts in the suspensions were therefore adjusted in an arbitrary fashion in these ratios: red blood cells 10, spleen cells 3, tumour cells 1. The viscosity of the medium will also affect the rate of settling. For this reason the suspending medium must be the same for all cell types. In the first set of experiments the cells were suspended in saline or in calcium free Locke's solution. This enabled a large range of concentrations of the agglutinating compounds to be tested without danger of causing precipitation of material from the medium itself. At the same time, tests were made on the amount of precipitation produced in serum over the same range of concentrations. This enabled the compounds to be tested later on cells suspended in serum, in the concentration range which did not cause precipitation of serum proteins. The use of serum enabled the cells to be examined under conditions approaching more nearly to those obtaining *in vivo*. The viability of the cells in the salt media and in serum was tested at various times by examination in the interference microscope and by the Lissamine green test (Goldacre, R. J., private communication). Healthy cells can be readily recognised in the interference microscope from the high density (concentration) of their cytoplasm ; the very early stages of lysis can also be easily recognised. The appearance of the outer membrane is also characteristic in healthy cells and can be observed in the interference microscope without the halo seen in the phase contrast microscope. In the Lissamine green test the dye is found to penetrate and stain the cells after death but does not penetrate healthy cells. In a freshly prepared suspension about 80 per cent of the cells were found to be viable and there appeared to be little difference between the viability of normal and of tumour cells. Cells which were kept at room temperature remained viable longer than those incubated at 36°–38°. Even after 24 hours a number of cells remained viable and after reheating to 38° C. were seen to be moving. In the experiments using saline, the final state of the suspension, after standing overnight, was recorded. But in the experiments with serum, observations were also made at successive short intervals of time because marked differences in the rates of precipitation of the various cell types were observed.

(c) *Methods of preparing cell suspensions*

As has been indicated, the malignant cell shows a degree of autonomy in as much as it has not been seen to establish permanent contacts under ciné micrographic examination and also carries a high negative charge. A degree of mechanical agitation might therefore be expected to separate malignant cells from the tumour stroma. The ascitic form of tumour may in fact represent an extreme case of the selection of more autonomous clones of increasing malignancy and decreasing adhesiveness. For the preparation of suspensions by agitation, small portions of the tumour are dissected out under asceptic conditions and are agitated by a sterile glass rod in the presence of the suspending fluid, which may be sterile saline or homologous serum. The agitation is carried out in a small screw-capped bottle. The suspension is then filtered through one thickness of sterile lint, mounted in a Seitz filter in lieu of a filter pad. The cell suspensions are then examined microscopically in order to ascertain viability and are counted in a haemocytometer slide. Blood suspensions for comparative purposes may be prepared by agitating freshly drawn blood with sterile glass beads in a screw-capped bottle, separating the fibrin, spinning down the red blood cells and resuspending in the ambient medium required.

Suspensions of other tissues may be obtained by a citrate perfusion technique (Anderson, 1953), using citrated Locke's solution injected into the aorta with severance of the appropriate afferent vessels. The perfused tissues, particularly liver, are very friable and readily suspend under the treatments indicated above. When this technique is used, the tumour tissue is also perfused in order that both cell types should receive comparable treatment. Finally, the suspension concentration is adjusted to the appropriate relative value (see above) and is ready for the experimental procedure. (For very short term experiments asepsis is not necessary.)

### EXPERIMENTAL RESULTS

The results obtained with various polyelectrolytes on red blood cells, Walker tumour cells and serum obtained from the same animals are shown in Fig. 1, 2 and 3. The successive stages of precipitation as seen in the Dreyer tubes were recorded as follows :

| | |
|---|---|
| Cloudiness . . . . | + |
| Cloudiness and precipitation on walls of tube . . . | ++ |
| Precipitation . . . | +++ |
| Dense precipitation . . | ++++ |

(a) *Summary of experiments using saline*

It can be seen from the results shown in Fig. 1, 2 and 3, that the compounds studied showed differences in their agglutinating power for tumour cells as com-

Fig. 1.—Graphs showing degree of agglutination of cells by various positively charged ions. Cells suspended in calcium-free Locke's solution. Agglutination measured according to arbitrary scale. + turbidity ; ++ deposition on walls of tube ; +++ settled precipitate ; ++++ dense precipitate.

pared with red blood cells, but no compound was found to show a preferential effect on tumours. In view of the encouraging results obtained in the earlier microscopic studies, the observations were extended to studies of cells suspended in homologous serum. The observations were also extended to include spleen cells. The results obtained are shown in Fig. 4.

FIG. 2.—Agglutination of cells by positively charged polymers. Cells suspended in calcium-free Locke's solution. Degree of agglutination to same scale as in Fig. 1.

FIG. 3.—Agglutinations of cells by positively charged polymers. Cells suspended in calcium-free Locke's solution. Degree of agglutination to same scale as in Fig. 1.

(b) *Summary of experiments using serum*

An examination of the curves shown in Fig. 4 indicates that the polyelectrolyte polyethylene imine shows a preferential adsorption on the tumour cells. In the concentration range 0·0015–0·0063 per cent agglutination of tumour cells occurs without any detectable effect on red blood cells or spleen cells. This effect is most marked if observations are made after a short interval of time.

(c) *The effect of heparin*

In view of the well known effect of heparin as an inhibitor of blood coagulation, this polymer was tested as an example of a *negatively* charged polyelectrolyte. No visible change occurred in cell suspensions in calcium free Locke's solution at final concentrations of heparin of 0·0015 per cent up to 0·5 per cent. But in the case of spleen cells the treated cells did in fact remain in suspension for a longer time than the controls, e.g. at 0·25 per cent heparin, after overnight standing

FIG. 4.—Agglutination of cells by positively charged ions. Cells suspended in rat serum. Degree of agglutination to same scale as in Fig. 1.

the cells had only settled half-way down the agglutination tube, whereas in the control they had settled to the bottom of the tube in the normal way; at 0·12 and 0·06 per cent the suspension was one-third of the way up the tube and at 0·03 per cent, one-quarter of the way up the tube. No effects were observed below a concentration of 0·03 per cent. Microscopic examination of the sample treated with 0·25 per cent heparin indicated that the small number of cell clumps observed in the control appeared to have been dispersed by heparin. Ten per cent of the cells were still viable in control and treated samples after standing overnight in this particular test.

(d) *Studies* in vivo

*In vivo* tests were carried out on mice bearing Ehrlich and Landschütz ascites tumours. Encouraging results were obtained but much work would be required to establish the best methods of administration. Polyethylene imine was the compound of choice in view of its preferential action on tumour cells.

In a typical experiment, a small tapping of ascitic fluid was taken from a 4-day implant of an Ehrlich ascites tumour. The fluid was examined under the interference microscope. Many active ascites cells could be seen. Occasional clumps of 3–4 cells were observed but no large aggregates could be detected. Very few blood cells were present. One hour later, 0·1 c.c. of a 0·5 per cent solution of polyethylene imine in saline was injected intraperitoneally. One hour later a second tapping was taken. Microscopic examination showed that the fluid was completely free of tumour cells but contained a number of unagglutinated blood cells. The few cells which remained appeared to be healthy. A lump of solid material was present in the tapping which was found to consist of a solid mass of agglutinated tumour cells. In another experiment a group of ten mice which had 1-day Ehrlich ascites implants were treated. Five were injected with solutions of polyethylene imine in saline over a period of six days. The control mice were given similar injections of saline. At the end of eight days, the mice were killed, all the ascitic fluid was withdrawn from each mouse and the volume measured. Each sample was then centrifuged under identical conditions and the cell volume measured. The results are shown in Table I.

TABLE I

| | Total Volume of ascitic fluid | | Volume of centrifuged cells | |
|---|---|---|---|---|
| Control mice | 7·0 | Mean 8·0 | 3·6 | Mean 4·2 |
| | 5·0 | | 3·0 | |
| | 11·4 | | 5·5 | |
| | 11·0 | | 5·5 | |
| | 5·5 | | 3·5 | |
| Treated mice | 3·0 | Mean 4·3 | 1·5 | Mean 1·5 |
| | 5·0 | | 1·7 | |
| | 5·0 | | 2·3 | |
| | — | | — | |
| | 4·0 | | 0·6 | |

The mean cell volume has been reduced to almost one-third by the treatment with polyethylene imine.

### DISCUSSION

The results described in the experimental section indicate that free cells in suspension are rapidly agglutinated in the presence of positively charged polyelectrolytes. Most of the positively charged polymers examined are effective at very low concentrations. The negatively charged polymer heparin does not produce agglutination nor does the unionised polymer, polysarcosine. This is to be expected in view of the higher negative charge carried by the tumour cells and by mammalian cells in general (Abramson, Moyer and Gorin, 1942). Neve, de Vries and Katchalsky (1955) have described the agglutination of red blood cells by polylysine and other positively charged polymers. The extraordinarily low concentration at which some of the polymers are effective (1 part in 7,000,000 in the case of polyvinyl pyridinium bromide) suggests strongly that a surface adsorption is involved. In this particular experiment, the concentration of polyvinyl pyridinium bromide was only sufficient to cover 1 per cent of the cell surface.

The increased adhesiveness of the tumour cells in the presence of polyelectrolytes (*P*) may be expected, because they have a greater probability of forming linkages between cells (*T*) than do the bivalent calcium ions

In most instances, the degree of agglutination reaches a maximum value (Fig. 1, 2 and 3) and dispersion occurs again at higher concentrations. Such an effect is also observed with simple colloidal systems and appears to be due to the formation of particles which are completely coated with polyelectrolytes. The coated particles are then soluble in a solution of similar molecules.

From the therapeutic point of view, the results indicate that some measure of tissue specificity resides in the cell surface. The increased charge on tumour cells is evidently not due to the appearance of a surface similar to a red blood cell. There are considerable differences in the relative affinities of the various polyelectrolytes for the various cell types. Although most polymers are more strongly absorbed by blood cells, one polyelectrolyte (polyethylene imine) shows some specificity towards the tumour in the presence of serum. Electrophoresis measurements of whole cells (Ambrose, James and Lowick, unpublished) indicate that this polymer at a concentration of 2 pts. in 10,000,000 reduces the mobility of kidney tumour cells without affecting normal kidney cells or red blood cells. At higher concentrations the polymer is also absorbed on the normal cells.

The degree of specificity so far observed is surprising in view of the simplicity of the chemical structures examined and suggests that a suitable charge distribution, ratio of hydrophilic to hydrophobic groups and spacial configuration of the polymer may lead eventually to the discovery of a compound with selective affinity for the tumour over a wide range of concentrations.

### SUMMARY

Previous evidence has indicated that the surface of cancer cells differs from that of normal cells and that these cells carry a higher negative electrical charge than those from which they are derived. Studies have therefore been made of the adsorption of positively charged polymers by these cells, as a possible method of reducing the surface charge. One polymer (polyethylene imine) has been found to show some specificity towards tumour cells.

The authors are grateful to Professor A. Haddow, F.R.S. for his encouragement in this work. They would also like to thank Professor F. Bergel and Professor J. A. V. Butler, F.R.S. for helpful advice and for the supply of materials for this investigation. They are grateful to Dr. R. J. Goldacre for biological advice in connection with the use of P.E.I. They are grateful to Dr. A. L. Walpole, Dr. P. Alexander and Dr. K. Stacey for supplies of polymers and to Mr. J. Carter for technical assistance.

This investigation has been supported by grants to the Chester Beatty Research Institute (Institute of Cancer Research : Royal Cancer Hospital) from the British Empire Cancer Campaign, Jane Coffin Childs Memorial Fund for Medical Research, the Anna Fuller Fund, and the National Cancer Institute of the National Institutes of Health, U.S. Public Health Service.

### REFERENCES

Abercrombie, M. and Ambrose, E. J.—(1955) *J. Photogr. Sci.*, **3**, 189.
*Idem* and Heaysman, J. E. M.—(1954) *Nature*, **174**, 697.
Abramson, H. A., Moyer, L. S. and Gorin, M. H.—(1942) ‘The Electrophoresis of Proteins’. New York (Reinhold), p. 44.
Ambrose, E. J., James, A. M. and Lowick, J.—(1956) *Nature*, **177**, 576.
Anderson, N. G.—(1953) *Science*, **117**, 627.
Huxley, J. S. and De Beer, G. R.—(1934) ‘The Elements of Experimental Embryology’. London (Cambridge University Press).
de Long, R. P., Coman, D. R. and Zeidman, I.—(1950) *Cancer*, **3**, 718.
Neve, A., de Vries, A. and Katchalsky, A.—(1955) *Biochim. biophys. Acta*, **17**, 538.